# Visualizing Potential Effects of Dentist Retirements on Accessibility to Dental Care Among Children in Alabama, 2019

**DOI:** 10.5888/pcd18.200410

**Published:** 2021-02-04

**Authors:** Steven Samsel, Ryan Tramp, Irem Sengul Orgut, Nickolas Freeman, Jason Parton, Matthew Hudnall, Dwight Lewis

**Affiliations:** 1 Culverhouse College of Business, The University of Alabama, Tuscaloosa, Alabama

**Figure:**
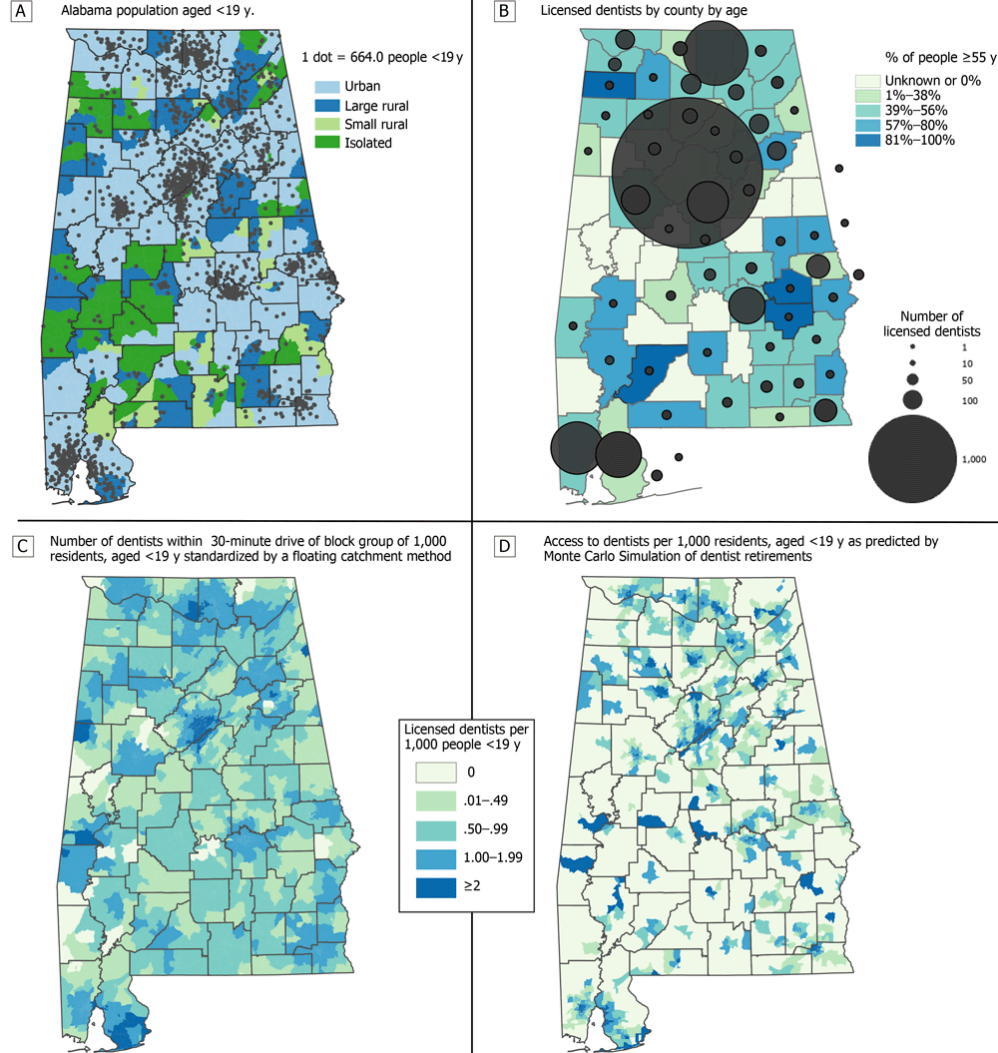
Four maps show the distribution of population and dentists in Alabama. Map A shows the distribution of the population aged 20 or younger; Map B, the distribution of licensed dentists by age across counties (counties with fewer than 3 dentists are not included); Map C, the number of dentists within a 30-minute drive of a block group of 1,000 residents aged 20 or younger, standardized by a floating catchment method; and Map D, shows access to dentists per 1,000 residents aged 20 or younger, as predicted by a Monte Carlo simulation of dentist retirements. Maps B and C include dentists’ data at the latitude and longitude point-level. Sources: 2018 American Community Survey 5-Year Estimates (8), Alabama Board of Dental Examiners (9), Rural Health Research Center (13), ESRI StreetMap Premium ArcGIS Pro version 2.5.0 (Esri), Python version 3.4 (Jupyter Project), and NAD 1983 HARN StatePlane Alabama West FIPS 0102 (Esri).

## Background

Regular dental visits can prevent dental problems (1,2). Under half of the US population aged 44 or younger is estimated to have untreated dental caries (3), and regular dental care during childhood can benefit oral health outcomes as an adult (2). Despite this evidence, access to dental care in the United States remains a challenge, especially among economically or socially marginalized groups (4). In 2018, only 230,490 of 736,103 (31.3%) beneficiaries under age 21 enrolled in Alabama’s Medicaid program used dental services (5). A crucial, but often overlooked barrier to dental accessibility in the United States is the aging of the dental workforce. In 2016, an estimated 40% of US dentists were aged 55 or older compared with 27% in 2001 (6). Data suggest that more than half of Alabama dentists are aged 50 or older (7), which indicates that a large number are expected to retire in the near future, which could result in a shortage of dentists.

The objective of our analysis was 2-fold. First, we aimed to highlight access to dentists among Alabamians aged 20 or younger in the context of evaluating a dental network adequacy policy that promotes access to dental care for all people aged 20 or younger, living 30 minutes or less of driving time from a licensed dentist. We then used national dentist retirement rates to describe the implications of such retirements on access to dental care. 

## Data and methods

Our study focuses on dental accessibility among young Alabama residents (<21 y) where each block group (n = 3,437) population count of young residents, as recorded in American Community Survey 5-year estimates of the 2018 US Census (8), was represented as the geometric center of its respective block group. We define accessibility as geospatial proximity to a state-licensed dentist in relation to a person’s home residence. The Alabama Board of Dental Examiners (9) provided 2020 data that was deidentified and geocoded at latitude and longitude point-levels. Statistics from the American Dental Association’s Health Policy Institute (7) were used to estimate the likelihood of a dentist retiring in the upcoming year, based on the age provided in the dental provider data (10). 

We used a 30-trial Monte Carlo simulation to simulate the effects of dentist retirements on access to dental care for residents aged 20 or younger. Similar to previous analyses (11), a 2-step floating catchment area method was employed to estimate accessibility to dentists in Alabama, and we used Monte Carlo methods to simulate future accessibility. We generated retirement scenarios that allowed us to assess the potential effect of dentist retirements on accessibility on the basis of the ages of currently practicing dentists and published retirement rates (10). Full systematic details on how this analysis was conducted with statistical formulas and Python code can be found at https://bit.ly/githubAccessBama.

Average differences and variances in accessibility estimates were observed in a simulation of dentist retirements to better understand differences in geospatial accessibility after accounting for the retirements. Comparisons of physical access to dental care by rurality augmented the retirement scenario. Rurality was operationalized by using the 2019 rural–urban commuting area codes from the Rural Health Research Center’s 4-level categorization (Rural Health Research Center). Automobile travel times were generated by using ESRI Streetmap Premium 2019 (Esri). All analyses were generated with ArcGIS Pro 2.5.0 (Esri) and Python 3.4 (Jupyter Project) by using multiple libraries. We used the Kruskal–Wallis test to examine differences in accessibility scores by rurality. Although findings presented in this article reflect modeling assumptions (eg, applying a drive-time catchment threshold of 30 minutes) used by the American Dental Association in an earlier study (11), interactive maps with the ability to manipulate various assumptions are available on a Tableau Software public dashboard (Supplemental file at https://public.tableau.com/shared/23ZDYJ77R).

## Highlights

The percentage of dentists who were likely to retire within the calendar year was 2.5% for those aged 34 or younger; 2.3%, 35 to 44; 4.0%, 45 to 54; 15.9%, 55 to 64; 40.9%, 65 to 74; 61.4%, 75 to 84; and 80.6%, 85 or older. On the basis of map analyses describing accessibility, we came to 3 conclusions. First, young people’s access to dentists appeared to be higher in Alabama urban areas than in rural areas (*P* < .001) (Table). The average accessibility score of an urban census block was about 1.28 dentists per 1,000 young people compared with about 0.85 dentists per 1,000 youths in rural areas. Second, considering our simulation of dentist retirements, rural regions on average would be more affected by retirements than urban regions. Third, although the retirement of aging dentists appeared, potentially, to affect various areas of Alabama, the southwest corner of the state appeared to be the most vulnerable. 

**Table:** Potential Effects of Dentist Retirements on Children in Alabama: Descriptives and Accessibility Scores^b^^,^ c ^,^ d by Rural Status^a^

Population	Urban	Large Rural	Small Rural	Isolated	Statewide
<20 y, n (%)	1,022,520 (78.5)	201,338 (15.5)	25,895 (2.0)	52,413 (4.0)	1,302,166 (100.0)
Block groups, n (%)	2,571 (74.8)	583 (17.0)	91 (2.6)	191 (5.6)	3,436 (100.0)
**Percentile ranked baseline accessibility scores, providers per 1,000 population aged <18 y**					
10th	0.46	0.44	0.32	0.25	0.43
25th	0.84	0.64	0.46	0.56	0.72
Median	1.28	0.80	0.64	0.77	1.12
75th	1.70	0.97	0.88	0.92	1.54
90th	2.24	1.42	1.09	1.32	2.11
**Percentile ranked retirement simulated accessibility scores, providers per 1,000 population aged <18 y**					
10th	0	0	0	0	0
25th	0.18	0	0	0	0.05
Median	0.71	0.70	0	0	0.66
75th	1.61	1.14	0.72	0.87	1.46
90th	2.58	1.90	1.26	1.79	2.41

a Rurality based on the Rural Health Research Center’s 4-Level Categorization at https://depts.washington.edu/uwruca/ruca-maps.php.

b Baseline accessibility scores calculated using a 2-step floating catchment area.

c Simulated accessibility scores calculated using a 30-trial Monte Carlo simulation of a 2-step floating catchment area.

d Details on this analysis, including formulas and Python code can be found at https://bit.ly/githubAccessBama.

Observation of the Tableau software public dashboard suggested that modifying the travel time threshold to operationalize access had a greater effect on young people in urban areas than young people in the rural southwestern and lower-central regions of Alabama. The high density of dentists working in urban regions most likely accounts for this difference. Although we focused on the outflow of dentists, some studies suggest that dental school graduates are more likely to seek employment in urban areas than in rural areas (12), which suggests that our results would be more pronounced if we included inflow estimation rates. Our maps and the online Tableau Software dashboard provide evidence that the potential retirement of aging dentists jeopardizes dental care access for young people in Alabama, especially those in rural areas. Stakeholders including the US Public Health Service (USPHS), the Alabama Medicaid Agency, and the Alabama Department of Public Health can utilize these preliminary findings to develop strategies for targeted investigations on possible clinical effects of this phenomenon. USPHS often provides incentives, such as scholarships and student loan forgiveness for enrolled clinicians willing to practice in underserved areas. The Alabama Medicaid Agency provides a significant amount of dental care to young people in Alabama, particularly those in rural areas where a large portion of citizens are enrolled in Medicaid. 

## Actions

Our study has limitations. First, only license information for dentists in Alabama were used in analyses. Young people in counties that border the neighboring states might choose to use the service of a dentist not licensed in Alabama. Our analyses, therefore, may have edge effect biases. Another limitation is that we focus on dentists retiring (outflow) and do not consider new dentists joining the workforce (inflow). We do this to provide a worst-case estimation of future dental care accessibility; however, future studies may also incorporate the inflow of dentists. Nonetheless, strengths in our analyses balance its limitations.

Our study is one of the few analyses in Alabama to assess the relationship between dentist age and access to dental care. To our knowledge, this is the first study to visualize the effect of dentist retirements on dental care accessibility, which has the potential to serve as a preliminary step in a planning management strategy for the allocation of dentists in areas of need. Institutions outside of Alabama can use our methods to estimate accessibility in their regions to examine the effects of key policy decisions before implementation.

## References

[1] American Dental Association. Action for dental health: bringing disease prevention into communities. a statement from the American Dental Association. 2013 https://www.ada.org/~/media/ADA/Public%20Programs/Files/bringing-disease-prevention-to-communities_adh.ashx. Accessed July 1, 2020.

[2] Achembong LN , Kranz AM , Rozier RG . Office-based preventive dental program and statewide trends in dental caries. Pediatrics 2014;133(4):e827–34. 10.1542/peds.2013-2561 24685954 PMC5002973

[3] National Center for Health Statistics. Oral and dental health. Centers for Disease Control and Prevention: US Department of Health and Human Services; 2017 https://www.cdc.gov/nchs/fastats/dental.htm. Accessed 2020 July 1, 2020.

[4] Bersell CH . Access to oral health care: a national crisis and call for reform. J Dent Hyg 2017;91(1):6–14. 29118145

[5] Alabama Medicaid Agency. Dental statistics FY 2010-2017. Montgomery, AL 2018 https://medicaid.alabama.gov/documents/4.0_Programs/4.2_Medical_Services/4.2.2_Dental/4.2.2_Dental_Stats_Report_5-3-17.pdf. Accessed July 1, 2020.

[6] Vujicic M . The “de-aging” of the dentist workforce. J Am Dent Assoc 2016;147(10):843–5. 10.1016/j.adaj.2016.06.016 27497867

[7] American Dental Association Health Policy Institute. Dentist profile snapshot by state: 2016. https://www.ada.org/~/media/ADA/Science%20and%20Research/HPI/Files/HPIData_Profile_2016.xlsx?la=en. Accessed July 1, 2020.

[8] US Census Bureau. American community survey 5-year estimates — geodatabase format 2020 https://www.census.gov/geographies/mapping-files/time-series/geo/tiger-data.html. Accessed July 1, 2020.

[9] Board of Dental Examiners of Alabama. (Licensing database not publicly available.) https://www.dentalboard.org/. Accessed December 28, 2021.

[10] Munson B , Vujicic M . Supply of full-time equivalent dentists in the US expected to increase steadily. Health Policy Institute research brief of the American Dental Association. July. 2018. http://www.ada.org/~/media/ADA/Science%20and%20Research/HPI/Files/HPIBrief_0718_1.pdf. Accessed December 7, 2020.

[11] Nasseh K , Eisenberg Y , Vujicic M . Geographic access to dental care varies in Missouri and Wisconsin. J Public Health Dent 2017;77(3):197–206. 10.1111/jphd.12197 28075494

[12] Alter D . Where have all the dentists gone? A question laboratory owners may be asking in the coming years. Aegis Dental Network. 2016 https://www.aegisdentalnetwork.com/idt/2016/12/trends-in-dentistry-where-have-all-the-dentists-gone. Accessed October 10, 2020.

[13] Rural Health Research Center. RUCA Data: code definitions: Version 2.0. University of Washington: Seattle, WA 2019 https://depts.washington.edu/uwruca/ruca-maps.php. Accessed July 1, 2020.

